# Intravoxel incoherent motion diffusion-weighted MR imaging parameters predict pathological classification in thymic epithelial tumors

**DOI:** 10.18632/oncotarget.17857

**Published:** 2017-05-15

**Authors:** Gang-Feng Li, Shi-Jun Duan, Lin-Feng Yan, Wen Wang, Yong Jing, Wei-Qiang Yan, Qian Sun, Shu-Mei Wang, Hai-Yan Nan, Tian-Yong Xu, Dan-Dan Zheng, Yu-Chuan Hu, Guang-Bin Cui

**Affiliations:** ^1^ Department of Radiology, Tangdu Hospital, Fourth Military Medical University, Xi’an, China; ^2^ Department of Pathology, Tangdu Hospital, Fourth Military Medical University, Xi’an, China; ^3^ MR Research China, GE Healthcare China, Beijing, China

**Keywords:** thymic epithelial tumor, intravoxel incoherent motion, DWI, masaoka stage, pathological type

## Abstract

We evaluated the performance of intravoxel incoherent motion (IVIM) parameters for preoperatively predicting the subtype and Masaoka stage of thymic epithelial tumors (TETs). Seventy-seven patients with pathologically confirmed TETs underwent a diffusion weighted imaging (DWI) sequence with 9 b values. Differences in the slow diffusion coefficient (D), fast perfusion coefficient (D), and perfusion fraction (f) IVIM parameters, as well as the multi b-value fitted apparent diffusion coefficient (ADC_mb_), were compared among patients with low-risk (LRT) and high-risk thymomas (HRT) and thymic carcinomas (TC), and between early stage (stages I and II) and advanced stage (stages III and IV) TET patients. ADC_mb_, D, and D values were higher in the LRT group than in the HRT or TC group, but did not differ between the HRT and TC groups. The mean ADC_mb_, D, and D values were higher in the early stage TETs group than the advanced stage TETs group. The f values did not differ among the groups. These results suggest that IVIM DWI could be used to preoperatively predict subtype and Masaoka stage in TET patients.

## INTRODUCTION

Although thymic epithelial tumors (TETs) are relatively rare, accounting for 0.2-1.5 % of all malignancies, they are the most common primary tumor of the anterior mediastinum [1, 2]. The major prognostic indicators for TETs are tumor invasiveness and histology, which is evaluated using the Masaoka staging system [3] and is an important indicator of candidacy for complete surgical resection. Optimal therapeutic strategies and prognoses for TETs differ depending on pathological type or stage [4], especially because surgery is not always the first step in treatment. For example, early stage (stages I and II) TETs are treated with surgery, while more advanced diseases (stages III and IV) require neoadjuvant chemotherapy [5–9]. It is therefore critical to accurately identify histological type and stage before treatment.

Imaging is an important noninvasive technique for the preoperative diagnosis, staging, and follow-up monitoring of TETs [10]. Conventional computed tomography (CT) and magnetic resonance imaging (MRI), which can provide detailed morphologic information regarding tumor location, size, shape, homogeneity, and other characteristics, are routinely used for imaging TET patients [11–13]. Although conventional imaging has also shown considerable potential, it relies on qualitative parameters and the presence of many overlapping features, and TET type and stage cannot be accurately assessed using conventional imaging [14].

Diffusion-weighted imaging (DWI), a noninvasive functional MRI technique, is considered the most sensitive method for detecting differences in the diffusion of molecular water within different tissues [15]. Apparent diffusion coefficient (ADC) values generated using DWI might be useful for quantitatively evaluating the pathological classification of TETs [16, 17]. Razek *et al*. reported that an ADC cutoff value of 1.22 ×10^−3^ mm^2^/sec was best for differentiating low risk thymoma (LRT) from high risk thymoma (HRT) and thymic carcinoma (TC), with a sensitivity of 87%, specificity of 85%, and accuracy of 86% [16]. Priola *et al*. found that the optimal threshold ADC value for differentiating LRT from HRT was 1.309 ×10^−3^ mm^2^/sec, with 94.7% diagnostic sensitivity, 63.6% specificity, and 78.1% accuracy [17]. However, an important limitation of DWI should be considered when interpreting these results. Perfusion due to the incoherent motion of blood in pseudorandom capillary networks can substantially confound diffusion measurements. Intravoxel incoherent motion (IVIM) based on DWI has been proposed as a way to overcome this limitation [18–20], and studies of human tumors, including brain, liver, pancreas, and bone lesions, have begun to demonstrate its efficacy [21–24]. However, whether IVIM DWI is effective in predicting the pathological classification of TETs and the specific parameters that should be applied in IVIM DWI analyses remain largely unknown.

In this study, we evaluated the diagnostic performance of IVIM DWI parameters in preoperatively predicting TET pathological subtypes and stages. In addition, we assessed the inter-observer variability of IVIM parameters.

## RESULTS

### Demographic data

The clinical and demographic characteristics of the patients are summarized in Table 1. The study group consisted of 53 males and 24 females with a mean age of 51.6 ± 12.4 years (range: 19 - 77 years). The major clinical presentations of the patients were myasthenia gravis (20.8%; 16 of 77 patients), chest pain (19.5%; 15 of 77), and respiratory symptoms (33.8%; 26 of 77); 4 (5.2%) patients presented with other symptoms, and the remaining 16 patients were without any discomfort (20.8%).

**Table 1:** Clinical and demographic characteristics of 77 TET patients

Patient characteristics	
**Age** (yrs)	
Mean ± SD	51.6 ± 12.4
Median	52.0
Range	19 - 77
**Sex - no. (%)**	
Males	53 (68.8)
Females	24 (31.2)
**Major symptoms or signs - no. (%)**	
No symptom	16 (20.8)
Myasthenia gravis	16 (20.8)
Chest pain	15 (19.5)
Respiratory symptoms	26 (33.8)
Other	4 (5.2)
**Method for obtaining pathologic results - no. (%)**	
Surgery	65 (84.4)
Puncture biopsy	12 (15.6)
**Masaoka-Koga Stage - no. (%)**	
Stage I	11 (14.3)
Stage II	22 (28.6)
Stage III	27 (35.1)
Stage IV	17 (22.1)
**WHO classification - no. (%)**	
A	3 (3.9)
AB	15 (19.5)
B1	5 (6.5)
B2	22 (28.6)
B3	9 (11.7)
Thymic Carcinoma	23 (29.9)
Squamous cell carcinoma	14 (18.2)
Adenocarcinoma	5 (6.5)
Neuroendocrine carcinomas	4 (5.2)

**Figure 1:**
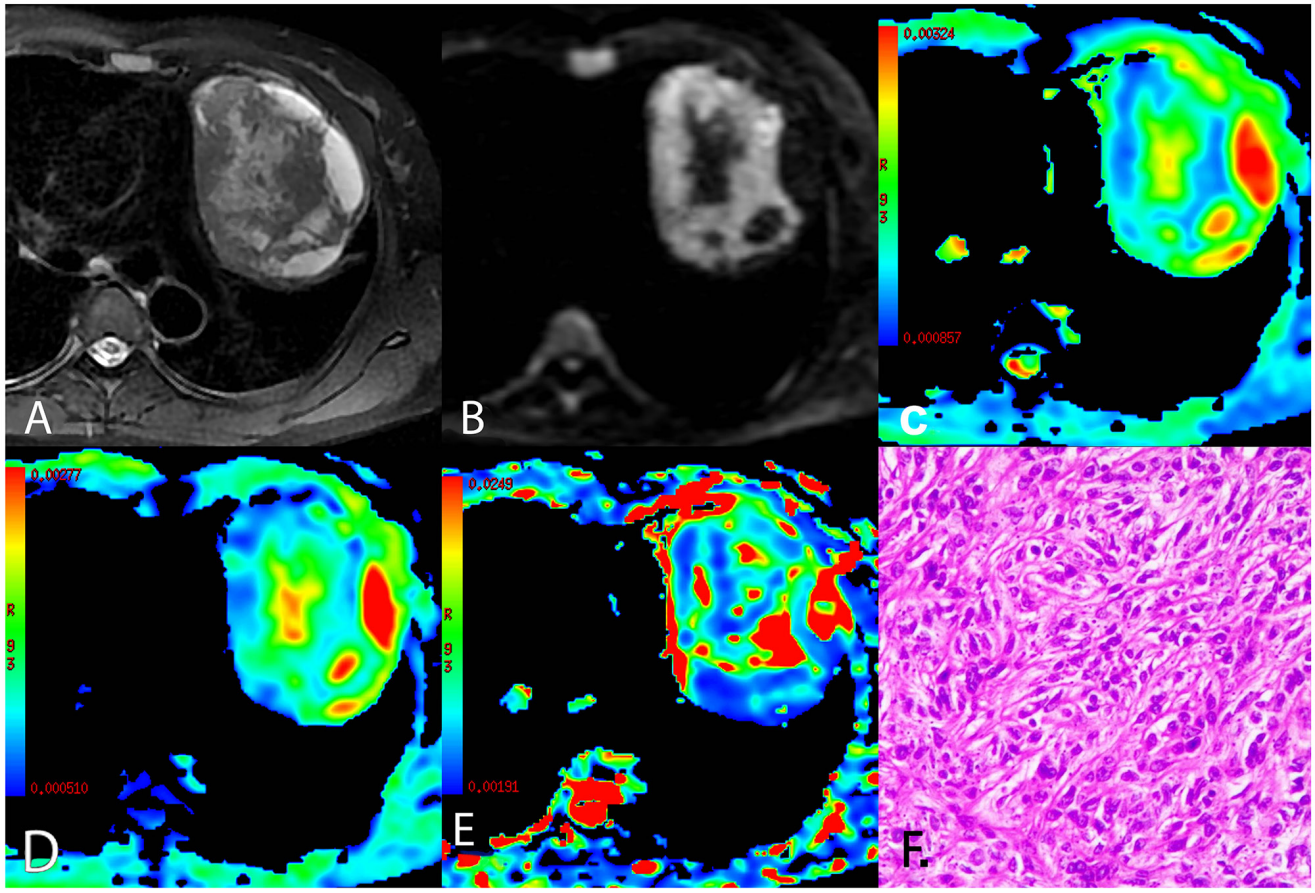
A representative case of low risk thymoma (type A) **(A)** Axial T2-weighted fat-suppressed MR image showing left-sided anterior mediastinal mass with an oval shape and smooth contours containing central cystic areas and peripheral cyst-like changes. **(B)** Diffusion-weighted trace image (b = 1000 sec/mm^2^) showing high signal intensity in solid parts and lower signal intensity in cystic areas of the tumor. **(C-E)** ADC_mb_ maps, D maps, and D* maps showing varying ADC_mb_, D, and D* values in different parts of the tumor (parametric values increase as color changes from dark blue to red). **(F)** Histological image showing diffuse growth of short spindle cells with bland nuclei (HE, 200 ×). ADC_mb_ = ADC calculated using mono-exponential model DWI (multi b-values: 0 - 1200 sec/mm^2^); D = ADC_slow_ or pure diffusion coefficient; D* = ADC_fast_ or pseudo-diffusion coefficient.

**Figure 2:**
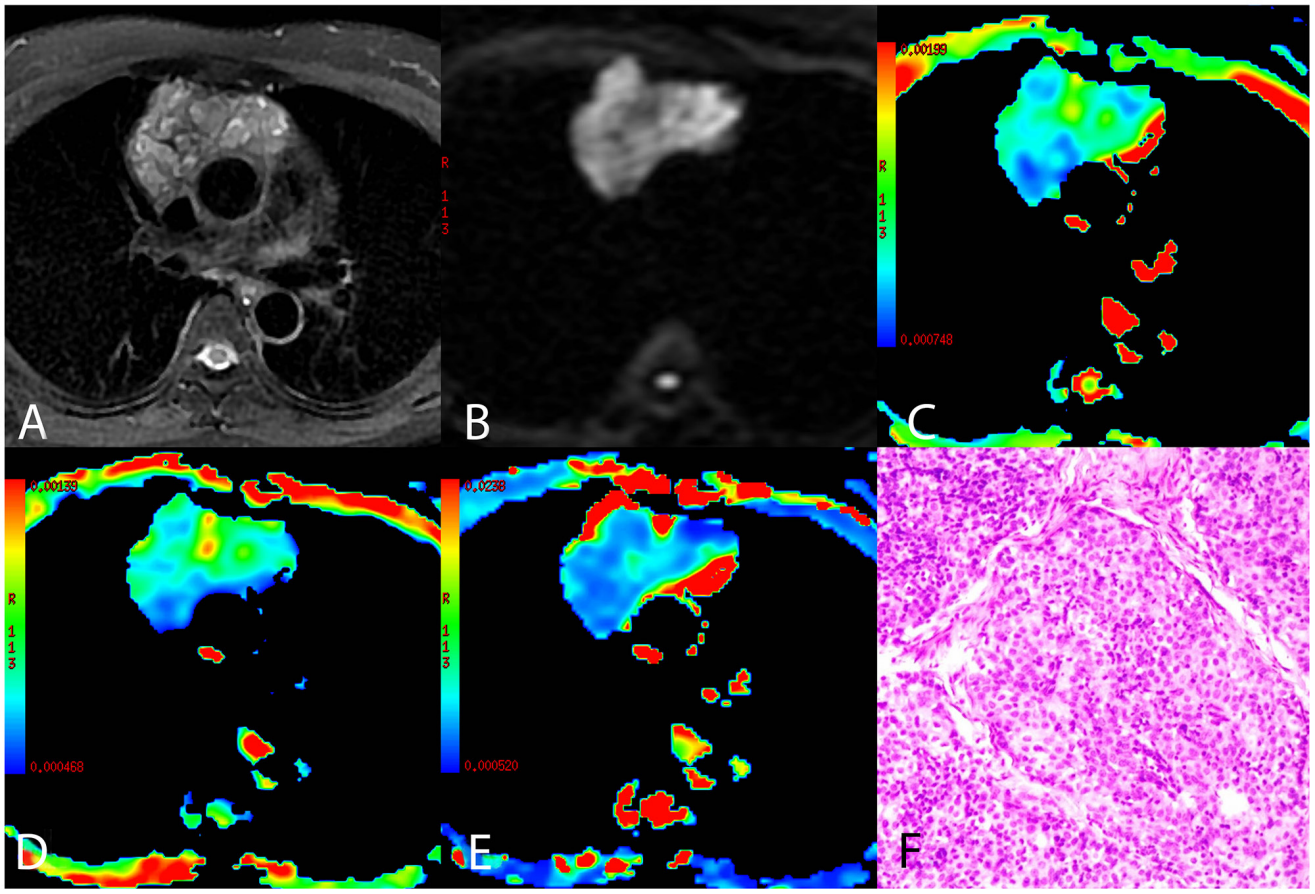
A representative case of high risk thymoma (type B3) **(A)** Axial T2-weighted fat-suppressed MR image showing an anterior mediastinal mass with an irregular shape and heterogeneous intensity. **(B)** Diffusion-weighted trace image (b = 1000 sec/mm^2^) showing higher intensity in the tumor compared to the normal chest wall muscle. **(C-E)** ADC_mb_ maps, D maps, and D* maps showing varying ADC_mb_, D, and D* values in different parts of the tumor (parametric values increase as color changes from dark blue to red). **(F)** Histological image showing epithelial cells arranged in dense sheets with some size variation and round nuclei (HE, 200 ×). ADC_mb_ = ADC calculated using mono-exponential model DWI (multi b-values: 0 - 1200 sec/mm^2^); D = ADC_slow_ or pure diffusion coefficient; D* = ADC_fast_ or pseudo-diffusion coefficient.

**Figure 3:**
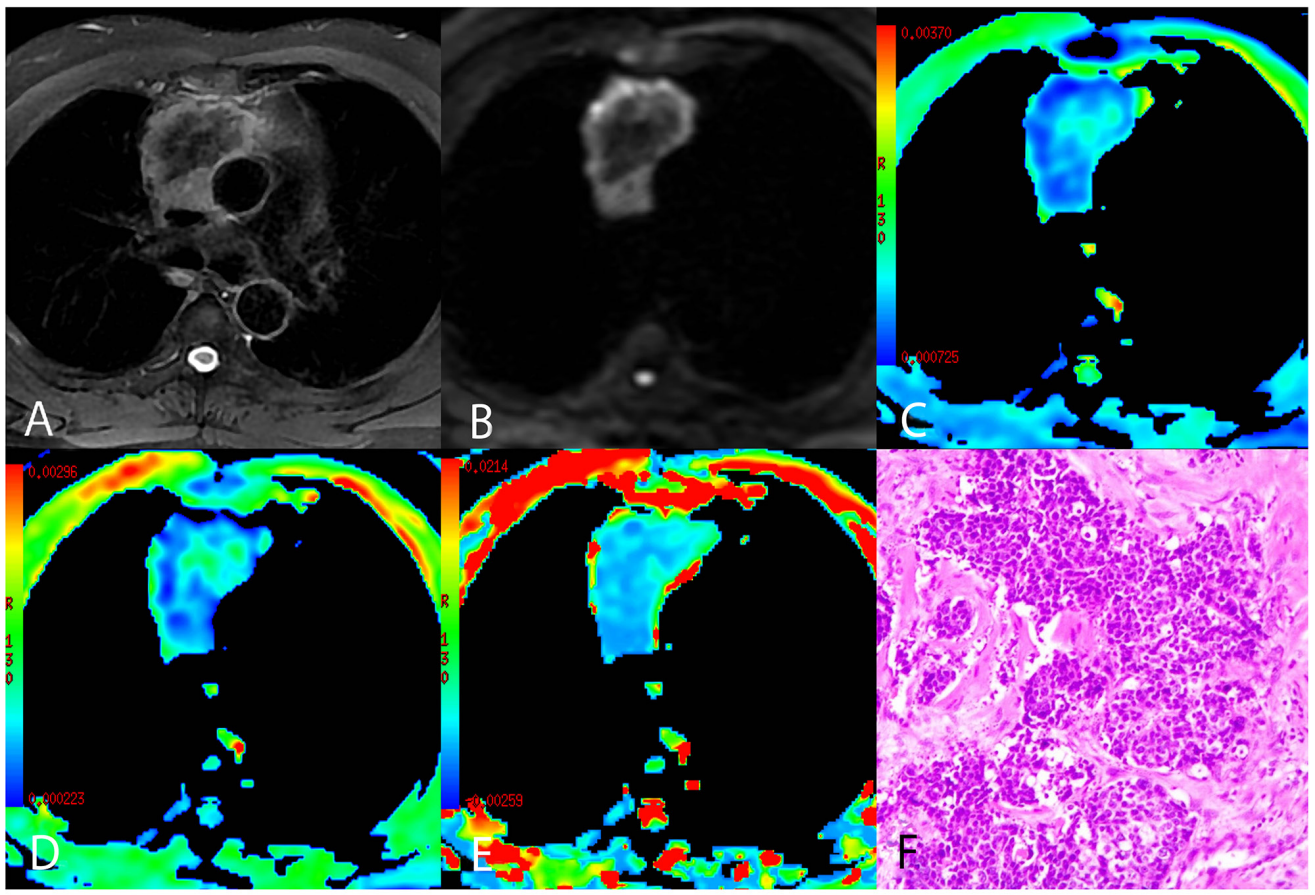
A representative case of thymic squamous cell carcinoma **(A)** Axial T2-weighted fat-suppressed MR image showing an anterior mediastinal mass with an irregular contour containing a low-signal area. **(B)** Diffusion-weighted trace image (b = 1000 sec/mm^2^) showing peripheral high-signal intensity and central low-signal intensity. **(C-E)** ADC_mb_ maps, D maps, and D* maps showing varying ADC_mb_, D, and D* values in different parts of the tumor (parametric values increase as color changes from dark blue to red). **(F)** Histological image showing carcinoma cells arranged in the nest with enlarged and atypical nuclei (HE, 200 ×). >ADC_mb_ = ADC calculated using mono-exponential model DWI (multi b-values: 0 - 1200 sec/mm^2^); D = ADC_slow_ or pure diffusion coefficient; D* = ADC_fast_ or pseudo-diffusion coefficient.

Sixty-five TET cases were staged based on surgical specimens, and the remaining 12 advanced stage patients were staged based on the presence of pleural or pericardium dissimilation or hematogenous metastasis at imaging and confirmatory puncture biopsies. Using the Masaoka clinical stages, 11 (14.3%) patients were classified as clinical stage I, 22 (28.6%) as stage II, 27 (35.1%) as stage III, and 17 (22.1%) as stage IV. The pathologic subtypes of the 77 TET patients were as follows: 23 patients had LRT (types A (n = 3), AB (n = 15), and B1 (n = 5)); 31 had HRT (types B2 (n = 22) and B3 (n = 9)); and 23 had TC (squamous cell carcinoma (n = 14), adenocarcinoma (n = 5), and neuroendocrine carcinomas (n = 4)). Representative histological images of LRT (type A), HRT (type B3) and TC (thymic squamous cell carcinoma) are shown in Figure 1F, Figure 2F, and Figure 3F, respectively.

Comparisons of conventional MRI findings depending on TET pathological type are shown in Table 2, and representative MR fat-suppressed T2-weighted images and diffusion-weighted images of LRT (type A), HRT (type B3), and TC (thymic squamous cell carcinoma) are shown in Figure 1A-B, Figure 2A-B, and Figure 3A-B, respectively. Overall, maximum tumor diameter, homogeneity, and the presence of pericardial or pleural effusion differed among patients with LRT, HRT, and TC (all *P* < 0.01), while mean tumor diameter and shape did not differ depending on TET subtype (*P* = 0.056 and 0.742, respectively).

**Table 2:** Comparison of conventional MRI findings for low- and high-risk thymoma and thymic carcinoma

Variable	LRT (n=23)	HRT(n=31)	TC (n=23)	*P* value
**Size**				
Maximum diameter - no. (%)				0.003*
< 6 cm	13 (56.5)	4 (12.9)	2 (8.7)	
6 - 9 cm	6 (26.1)	15 (48.4)	13 (56.5)	
≥ 9 cm	4 (17.4)	12 (38.7)	8 (34.8)	
Mean diameter (mean ± sd; cm)	5.62 ± 2.35	6.33 ± 1.65	6.99 ± 1.45	0.056^#^
**Shape - no. (%)**				0.742
Round	6 (26.1)	12 (38.7)	9 (39.1)	
Oval	11 (47.8)	14 (45.2)	11 (47.8)	
Plaque	6 (26.1)	5 (16.1)	3 (13.0)	
**Homogeneity - no. (%)**				<0.001
Heterogeneous	2 (8.7)	22 (71.0)	11 (47.8)	
High-signal foci	17 (73.9)	4 (12.9)	2 (8.7)	
Low-signal foci	4 (17.4)	5 (16.1)	10 (43.5)	
**Pericardial or pleural effusion - no. (%)**				<0.001
Yes	2 (8.7)	8 (25.8)	14 (60.9)	
No	21 (91.3)	23 (74.2)	9 (39.1)	

* Calculated using Kruskal-Wallis test;^#^calculated using Welch test; LRT = low-risk thymoma; HRT = high-risk thymoma; TC = thymic carcinoma.

### Comparison of parameters in low-risk (LRT) and high-risk thymomas (HRT) and thymic carcinomas (TC) patients for both readings

**Figure 4:**
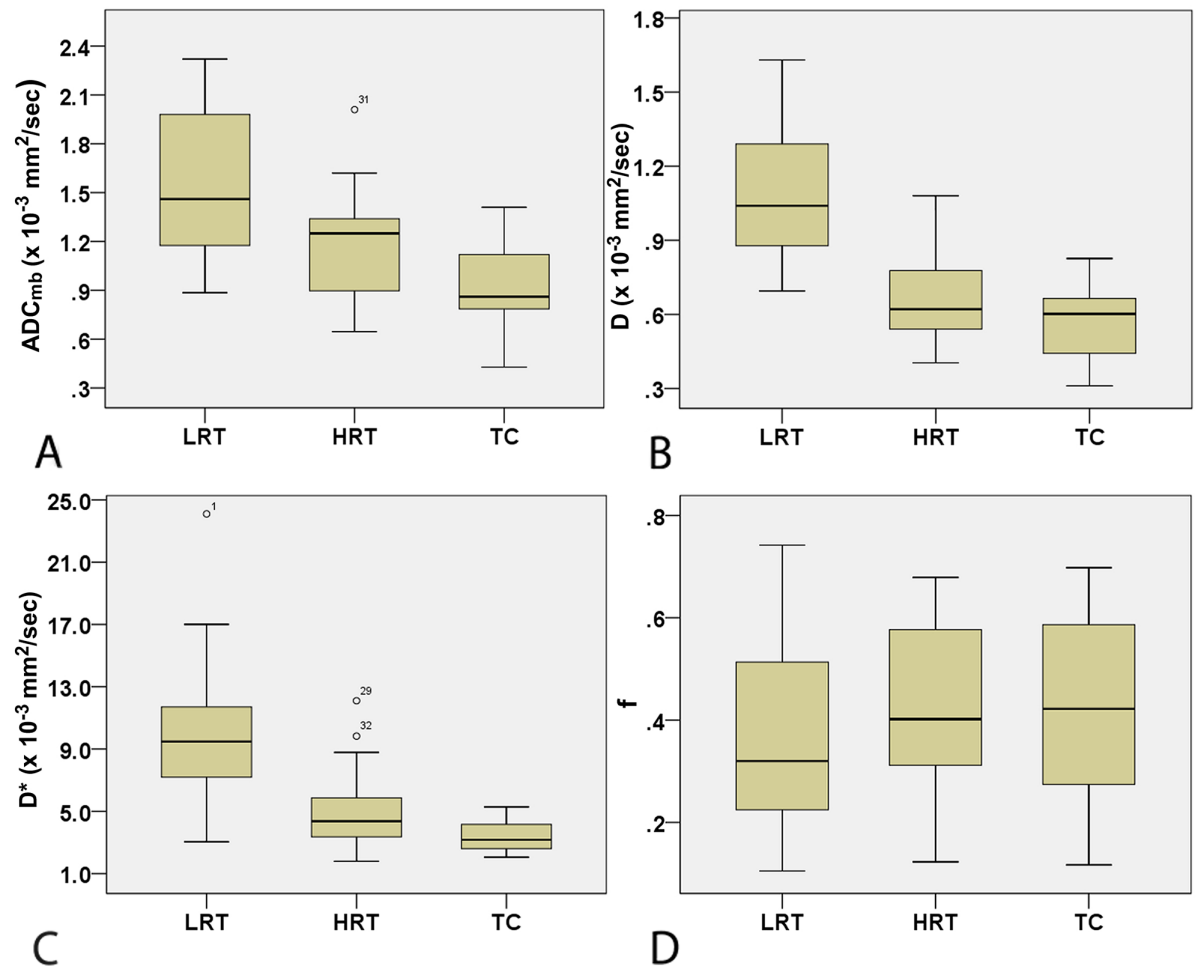
Box plots for ADC_mb_ **(A)**, D **(B)**, D* **(C)**, and f **(D)** valuesin LRT, HRT, and TC patients. LRT = low risk thymoma; HRT = high risk thymoma; TC = thymic carcinoma; ADC_mb_ = ADC calculated using mono-exponential model DWI (multi b-values: 0-1200 sec/mm^2^); D = ADC_slow_ or pure diffusion coefficient; D* = ADC_fast_ or pseudo-diffusion coefficient; f = perfusion fraction.

Comparisons of the ADC_mb_ and IVIM parameters among patients with LRT, HRT, and TC are shown in Table 3. A one-way ANOVA revealed that the mean ADC_mb_, D, and D* values in both readings were higher in the LRT group than in the HRT and TC groups (ADC_mb_: 1.55, 1.17, and 0.94 × 10^−3^ mm^2^/sec in the first reading (Figure 4A) and 1.56, 1.23, and 0.96 ×10^−3^ mm^2^/sec in the second reading; D: 1.09, 0.66, and 0.57 × 10^−3^ mm^2^/sec in the first reading (Figure 4B) and 1.14, 0.69, and 0.57 × 10^−3^ mm^2^/sec in the second reading; D*: 10.06, 4.93, and 3.35 × 10^−3^ mm^2^/sec in the first reading (Figure 4C) and 10.47, 5.29, and 3.90 × 10^−3^ mm^2^ /sec in the second reading, respectively; all *P* < 0.001); ADC_mb_ and D values did not differ between the HRT and TC groups in either reading (first reading, *P* = 0.018 and 0.128 and second reading, *P* = 0.021 and 0.042 for ADC_mb_ and D, respectively). In addition, the f value did not differ among any of the groups in either reading (*P* > 0.05) (Figure 4D). Representative ADC_mb_, D, and D* maps for patients with LRT (type A), HRT (type B3), and TC (thymic squamous cell carcinoma) are shown in Figure 1C-E, Figure 2C-E, and Figure 3C-E, respectively.

**Table 3:** Comparison of IVIM parameters among low- and high-risk thymoma and thymic carcinoma

Parameters	LRT (n = 23)	HRT (n = 31)	TC (n = 23)	*P* value
**First reading**				
ADC_mb_ (×10^−3^ mm^2^/sec)	1.55 ± 0.46 ^a^	1.17 ± 0.31 ^b^	0.94 ± 0.26 ^b^	< 0.001
D (×10^−3^ mm^2^/sec)	1.09 ± 0.26^a^	0.66 ± 0.19^b^	0.57 ± 0.15^b^	< 0.001
D* (×10^−3^ mm^2^/sec)	10.06 ± 4.51^a^	4.93 ± 2.28^b^	3.35 ± 0.95^c^	< 0.001
f	0.36 ± 0.18	0.42 ± 0.15	0.44 ± 0.18	0.239
**Second reading**				
ADC_mb_ (×10^−3^ mm^2^/sec)	1.56 ± 0.44^a^	1.23 ± 0.33^b^	0.96 ± 0.27^b^	< 0.001
D (×10^−3^ mm^2^/sec)	1.14 ± 0.31^a^	0.69 ± 0.20^b^	0.57 ± 0.15^b^	< 0.001
D* (×10^−3^ mm^2^/sec)	10.47 ± 5.49^a^	5.29 ± 2.23^b^	3.90 ± 1.14^c^	< 0.001
f	0.43 ± 0.21	0.47 ± 0.15	0.43 ± 0.17	0.603

The data are expressed as means ± standard deviation*. P* < 0.05 indicated a statistically significant difference among groups based on one-way ANOVA. The different letters represent significant differences between two groups based on post-hoc tests (*P* < 0.05/3).

LRT = low-risk thymoma; HRT = high-risk thymoma; TC = thymic carcinoma; ADC_mb_ = ADC calculated using mono-exponential model DWI (multi b-values: 0-1200 sec/mm^2^); D = ADC_slow_ or pure diffusion coefficient; D* = ADC_fast_ or pseudo-diffusion coefficient; f = perfusion fraction.

### Comparison of parameters in early and advanced stage TETs

**Figure 5:**
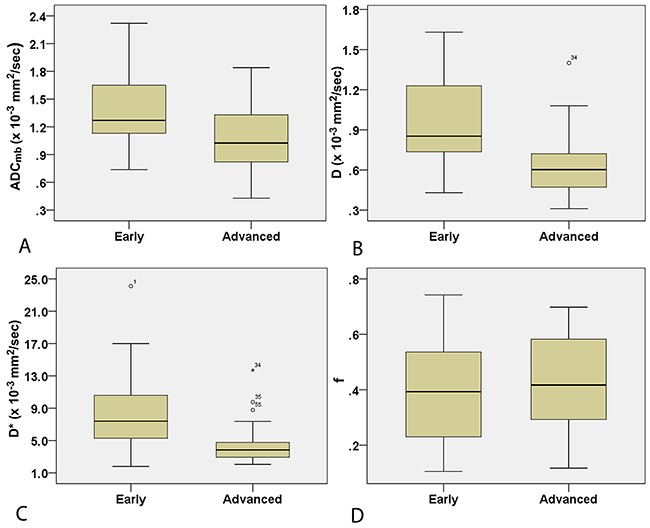
Box plots for ADC_mb_ **(A)**, D **(B)**, D* **(C)**, and f **(D)** values in early and advanced stage TETs. ADC_mb_ = ADC calculated using mono-exponential model DWI (multi b-values: 0-1200 sec/mm^2^); D = ADC_slow_ or pure diffusion coefficient; D* = ADC_fast_ or pseudo-diffusion coefficient; f = perfusion fraction.

Comparisons of IVIM parameters between early and advanced stage TETs groups are shown in Table 4. The mean ADC_mb_, D, and D* values in both readings were higher in the early stage group than in the advanced stage group (ADC_mb_: 1.41 vs. 1.06 × 10^−3^ mm^2^/sec in the first reading (Figure 5A) and 1.47 vs. 1.08 × 10^−3^ mm^2^/sec in the second reading; D: 0.93 vs. 0.64 × 10^−3^ mm^2^/sec in the first reading (Figure 5B) and 1.00 vs. 0.63 × 10^−3^ mm^2^/sec in the second reading; D*: 8.19 vs. 4.34 × 10^−3^ mm^2^/sec in the first reading (Figure 5C) and 8.62 vs. 4.77 × 10^−3^ mm^2^/sec in the second reading; all *P* < 0.05). The f values did not differ between groups in either reading (*P* > 0.05) (Figure 5D).

**Table 4:** Comparison of IVIM parameters between early and advanced stage TETs

Parameters	Early stage (n = 33)	Advanced stage (n = 44)	t	*P* value
**First reading**				
ADC_mb_ (×10^−3^ mm^2^ /sec)	1.41 ± 0.46	1.06 ± 0.31	3.747	< 0.001
D (×10^−3^ mm^2^/sec)	0.93 ± 0.30	0.64 ± 0.23	4.708	< 0.001
D* (×10^−3^ mm^2^ /sec)	8.19 ± 4.70	4.34 ± 2.24	4.346	< 0.001
f	0.38 ± 0.18	0.43 ± 0.17	1.188	0.239
**Second reading**				
ADC_mb_ (×10^−3^ mm^2^ /sec)	1.47 ± 0.45	1.08 ± 0.31	4.218	< 0.001
D (×10^−3^ mm^2^/sec)	1.00 ± 0.34	0.63 ± 0.20	5.581	< 0.001
D* (×10^−3^ mm^2^ /sec)	8.62 ± 5.50	4.77 ± 1.91	3.846	< 0.001
f	0.46 ± 0.19	0.44 ± 0.16	0.460	0.647

The data are expressed as means ± standard deviation. ADC_mb_ = ADC calculated using mono-exponential model DWI (multi b-values: 0 - 1200 sec/mm^2^); D = ADC_slow_ or pure diffusion coefficient; D* = ADC_fast_ or pseudo-diffusion coefficient; f = perfusion fraction.

### Analysis of diagnostic efficacy of the first reading

**Figure 6:**
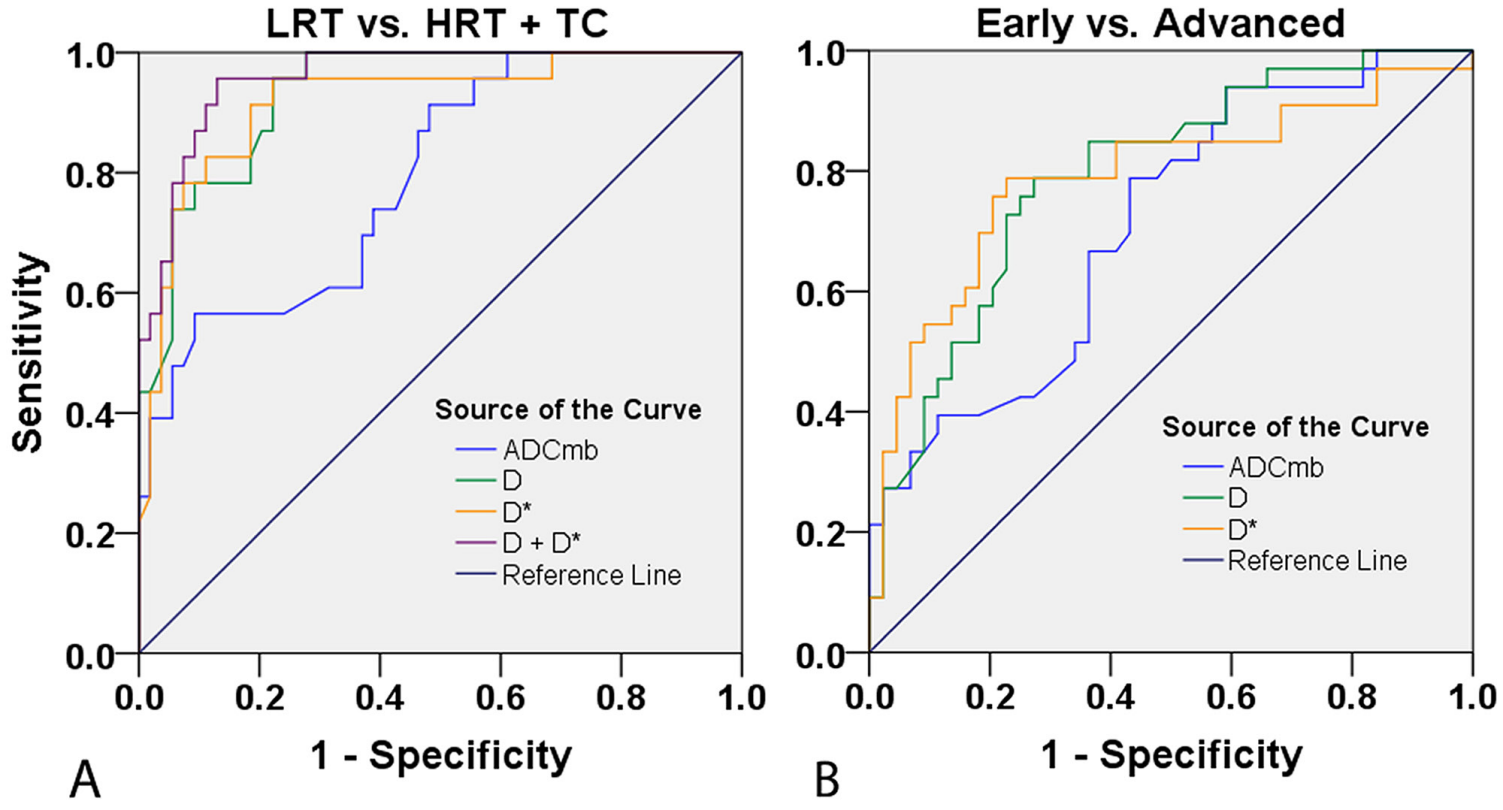
Receiver operating characteristic (ROC) curves for differentiating the performance of IVIM parameters in patients with different TET types based on WHO classification and Masaoka-Koga stage **(A)** LRT vs. HRT and TC based on ADC_mb_, D, and D* values. **(B)** Early vs. advanced stage based on ADC_mb_, D, and D* values. LRT = low risk thymoma; HRT = high risk thymoma; TC = thymic carcinoma; ADC_mb_ = ADC calculated using mono-exponential model DWI (multi b-values: 0-1200 sec/mm^2^); D = ADC_slow_ or pure diffusion coefficient; D* = ADC_fast_ or pseudo-diffusion coefficient.

In ROC analyses, the D value achieved a higher diagnostic efficacy, with an AUC of 0.933, 95.7% sensitivity, 77.8% specificity, 83.1% accuracy, 64.7% positive predictive value (PPV), and 97.7% negative predictive value (NPV) for differentiating LRT from HRT and TC at the cutoff value of 0.747 × 10^−3^ mm^2^/sec. The AUC, sensitivity, specificity, accuracy, PPV, NPV, and cutoff values for differentiating LRT from HRT and TC were 0.793, 56.5 %, 90.7 %, 79.2 %, 70.6 %, 81.7 %, 1.415 × 10^−3^ mm^2^/sec for ADC_mb_ and 0.919, 95.7 %, 77.8 %, 83.1 %, 64.7 %, 97.7 %, and 5.256 ×10^−3^ mm^2^/sec for D*, respectively. We then used a binary logistic regression with group as dependent variable and using D and D* as covariates to acquire *P* values for each patient, which were used for ROC analyses. Logistic (D, D*) achieved the highest diagnostic efficacy with an AUC of 0.959, 95.7% sensitivity, 87.0% specificity, 89.6% accuracy, 75.9% PPV, and 97.9% NPV (Table 5 and Figure 6A).

**Table 5:** Comparisonof the diagnostic efficacy of IVIM parameters in differentiating TETs based on WHO classification and Masaoka-Koga stage and comparisons of TET ADCs with published data

Parameters	AUC	Sensitivity (%)	Specificity (%)	Accuracy (%)	PPV (%)	NPV (%)	Cutoff value
**LRT vs. HRT+TC**							
ADC_mb_ (×10^−3^ mm^2^/sec)	0.793	56.5	90.7	79.2	70.6	81.7	1.415
D (×10^−3^ mm^2^/sec)	0.933	95.7	77.8	83.1	64.7	97.7	0.747
D* (×10^−3^ mm^2^/sec)	0.919	95.7	77.8	83.1	64.7	97.7	5.256
Logistic (D, D*) ^#^	0.959	95.7	87.0	89.6	75.9	97.9	0.193
ADC (×10^−3^ mm^2^/sec) (16)*	0.851	87.0	85.0	86.0	87.0	85.0	1.22
ADC (×10^−3^ mm^2^/sec) (17)^§^	0.864	94.7	63.6	78.1	−	−	1.309
**Early vs. advanced stage**							
ADC_mb_ (×10^−3^ mm^2^/sec)	0.711	78.8	56.8	64.9	56.5	77.4	1.095
D (×10^−3^ mm^2^/sec)	0.793	78.8	72.7	75.3	68.4	82.1	0.694
D* (×10^−3^ mm^2^/sec)	0.789	78.8	77.3	77.9	72.2	82.9	4.88
ADC (×10^−3^ mm^2^/sec) (17)^§^	0.730	91.7	58.8	73.2	−	−	1.243

AUC = area under curve; PPV = positive predictive value; NPV = negative predictive value; LRT = low risk thymoma; HRT = high risk thymoma; TC = thymic carcinoma; ADC_mb_ = ADC calculated using mono-exponential model DWI (multi b-values: 0 - 1200 sec/mm^2^); D = ADC_slow_ or pure diffusion coefficient; D* = ADC_fast_ or pseudo-diffusion coefficient.

^#^The results of Logistic (D, D*) were acquired using group as the dependent variable and the D and D* parameters as covariates to generate a binary logistic regression and *P* values for each patient. This *P* value was analyzed using ROC. The actual Logistic (D, D*) for LRT vs. HRT+TC model was as follows: ln[P/(1-P)] = 9.364 - 6.763D - 0.446D*.

*Results from Razek *et al*. for differentiating LRT from HRT+TC (16).

^§^Results from Priola *et al*. for differentiating LRT from HRT (17).

The AUC, sensitivity, specificity, accuracy, PPV, NPV, and cutoff values for differentiating early from advanced stages of TETs for ADC_mb_, D and D* were as follows: ADC_mb_, 0.711, 78.8 %, 56.8 %, 64.9 %, 56.5 %, 77.4 %, and 1.095 × 10^−3^ mm^2^/sec; D, 0.793, 78.8 %, 72.7 %, 75.3 %, 68.4 %, 82.1 %, and 0.694 × 10^−3^ mm^2^/sec; and D*, 0.789, 78.8 %, 77.3 %, 77.9 %, 72.2 %, 82.9 %, and 4.88 × 10^−3^ mm^2^/sec (Table 5 and Figure 6B).

### Inter-reader variability for ADC_mb_, D, D*, and f values

As shown in Table 6, the inter-observer ICC value for ADC_mb_ was close to 1 (0.917, *P* < 0.001), and the ICC values for D and D* were higher than 0.75 (0.821 and 0.786, respectively, *P* <0.001), suggesting that these quantitative MRI parameters could be measured with a high degree of reliability. In contrast, inter-observer ICC for f values was 0.662, indicating relatively poor reliability for this measure.

**Table 6:** Reliability analysis between the first and second parameter measurements

Parameters	ICC	*P* value	95% CI
ADC_mb_ (×10^−3^ mm^2^/sec)	.917	< 0.001	0.873 - 0.947
D (×10^−3^ mm^2^/sec)	.821	< 0.001	0.732 - 0.882
D* (×10^−3^ mm^2^/sec)	.786	< 0.001	0.682 - 0.858
f (%)	.662	< 0.001	0.515 - 0.771

ICC = intraclass correlation; 95% CI = 95% confidence interval; ADC_mb_ = ADC calculated using mono-exponential model DWI (multi b-values: 0 - 1200 sec/mm^2^); D = ADC_slow_ or pure diffusion coefficient; D* = ADC_fast_ or pseudo-diffusion coefficient; f = perfusion fraction.

## DISCUSSION

In the present study, we evaluated whether IVIM DWI predicted the histological type and Masaoka stage of TETs. We found that the ADC_mb_, D, and D* parameters differed between patients with LRT and those with HRT or TC. We also determined optimal cutoff values for and analyzed the reliability of these parameters to determine their potential utility in clinical evaluation of TETs before treatment.

Conventional MRI parameters are helpful in differentiating different TET subtypes [13, 16, 25]. As in previous studies, we found here that low-signal foci on T2WIs and pericardial or pleural effusion were more common in TCs than in low- and high-risk thymomas, and high-signal foci on T2WIs were more common in LRTs. In addition, the maximum diameters of LRTs were smaller than those of HRTs and TCs.

DWI has also been used to differentiate the different TET histological types and clinical stages [16, 17]. Enlarged nuclei, hyperchromatism, angulation of nuclear contour, and hypercellularity, in which the extracellular matrix and intra- and extracellular diffusion space for water molecules, and in turn ADC values, are reduced due to histologic characteristics, occur more frequently in HRT, TC, and advanced stage TETs than in LRT and early stage TETs [16, 17]. Similarly, we also detected a decrease in ADC_mb_ and D values in HRT, TC, and advanced stage TETs compared to LRT and early stage TETs. Interestingly, we confirmed that the D value achieved a higher diagnostic efficacy than the ADC_mb_ value in differentiating LRT from HRT and TC. It is possible that the D value reflects pure molecular diffusion more accurately than other measures. However, both D and ADC_mb_ might be useful as predictive markers when evaluating TETs.

A previous study identified a correlation between tumor angiogenesis and invasiveness in TET patients [26]. Theoretically, the D* value derived from the IVIM model is influenced by microvessel density (MVD) within the tumor and should be higher in individuals with higher risk or more advanced tumor stages [27]. In contrast, our results suggest that D* values were higher in those with LRT and early stage TETs than in those with HRT or TC and advanced stage TETs. Interestingly, these findings are consistent with the results of a CT contrast enhancement study in which maximal contrast-enhanced ranges (CE_max_) were higher in low risk subtypes of thymoma (type A and AB) than in other TETs [11]. Similarly, this unique perfusion or blood supply feature of TETs may be explained by a study which demonstrated that the short-spindled variant (57% histologic patterns of thymoma type A and AB) was composed of oval to short spindle cells typically arranged in a hemangiopericytic or microcystic pattern [28]. Those findings, together with our results, indicate that D* might be valuable for accurate prediction of TET type and stage.

Interestingly, f values did not differ among the different patient groups. Acquiring multiple b value DWIs of the chest remains technically challenging, and images may be influenced by motion artifacts related to breathing and cardiovascular pulsation and susceptibility artifacts associated with air-tissue interfaces [15]; here, we used an echo planar imaging (EPI) sequence with array spatial sensitivity encoding technique (ASSET) and respiratory trigger to reduce these artifacts. However, additional studies are needed to examine these issues.

In this study, we also evaluated the diagnostic efficacy of IVIM parameters in differentiating TET types and stages. D and D*, with AUCs of 0.933 and 0.919, respectively, performed relatively well, while the binary logistic regression analysis of both D and D* had the highest efficacy, with an AUC of 0.959, in differentiating LRT from HRT and TC. IVIM DWI may therefore be a useful supplement to conventional MRI parameters in TET typing.

As in a previous study [17], we also found that neither ADC_mb_ nor IVIM parameters performed particularly well in differentiating early from advanced TETs. However, TETs of all types can follow an aggressive clinical course [2], which might explain at least in part this relatively low performance.

An ICC analysis was performed to examine the reliability of the measurements performed by two independent radiologists in this study. The ICC value for ADC_mb_ was close to 1 (0.917), and the ICC value for D was higher than 0.75 (0.821), suggesting that the measurements were reliable. However, the ICC values of 0.786 for D* and 0.662 for f reveal that consistency in the measurement of these two parameters was relatively poor.

Both full and segmented fitting algorithms are frequently used in studies employing IVIM. The results of a recent study suggest that segmented fitting, which was used here, is preferable to full fitting, especially when the number of b-values is limited and SNR is lower [29]. Unlike several previous studies, we used D* rather than D+D* in first exponent of equation 1 in our IVIM model. We justify this approximation by noting that, because D* is usually larger than D by an order of magnitude, the slow diffusion represented by D contributes very little to the signal when b values are low.

Some limitations of this study should be considered when interpreting the results. First, we did not compare our findings to tumor perfusion data acquired using dynamic contrast enhanced images. Second, twelve advanced stage TET patients did not undergo surgery owing to widespread invasion or hematogenous metastasis identified by puncture biopsies and staged based on imaging findings; this may have introduced bias into our analysis. Third, ROIs were drawn manually, which might have introduced a sampling bias; the application of histogram analysis may improve diagnostic efficacy in future studies. Finally, negative results were obtained for some f value measurements; the meaning of these values is unclear and should be examined further.

In conclusion, our results suggest that several parameters from IVIM DWI, including ADC_mb_, D, and D*, may be useful in predicting TET pathological classification and, together with routine MRI parameters, might help surgeons preoperatively stage TET patients.

## MATERIALS AND METHODS

### Subjects

**Figure 7:**
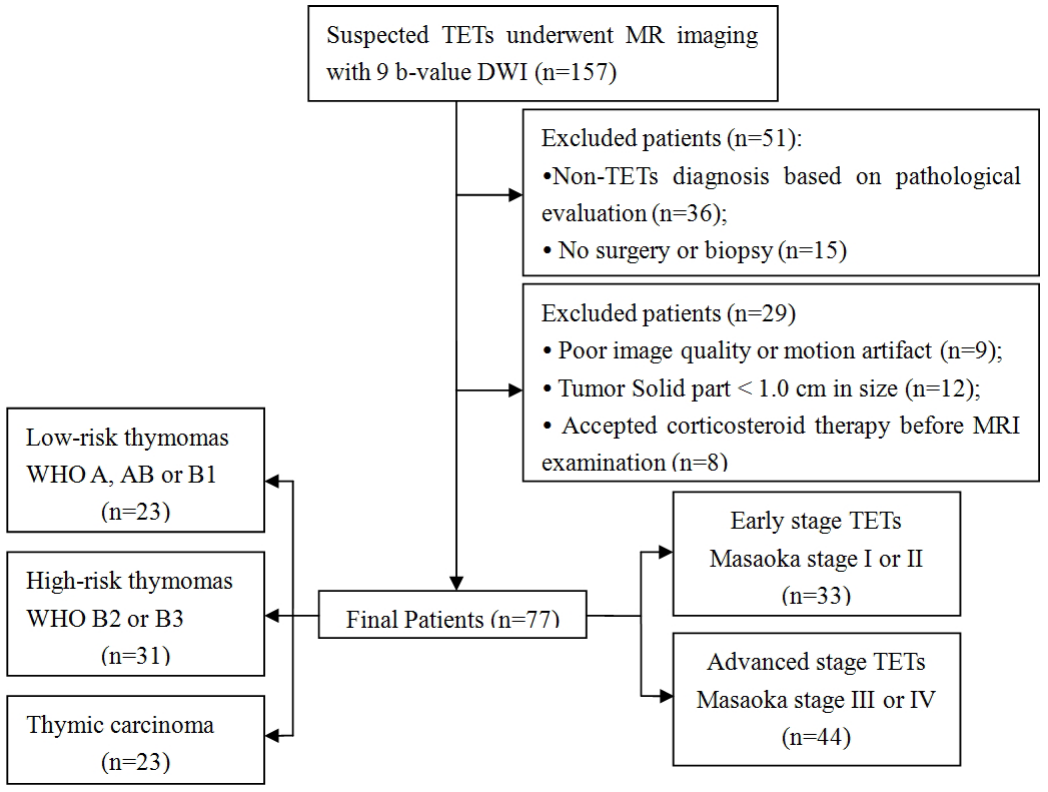
Flow diagram showing patient selection strategy and inclusion and exclusion criteria

This retrospective single-center study was approved by the Ethics Committee of Tangdu Hospital of the Fourth Military Medical University, and informed consent was waived. The study was conducted in accordance with the Declaration of Helsinki. Between December 2014 and May 2016, 157 consecutive patients with suspected TETs underwent routine MRI evaluation and IVIM DWI of the chest. Of these, 36 patients who were not diagnosed with TETs based on pathological evaluation, 15 patients for whom neither surgery nor biopsy were performed, 9 patients for whom images were of poor image quality or had motion artifacts, 12 patients with solid tumor portions < 2.0 cm in size, and 8 patients who received corticosteroid therapy before MRI examination were excluded. The final study population was comprised of 77 patients (53 men, 24 women; mean age, 52 years; age range, 19-77 years) newly diagnosed with TETs according to the inclusion and exclusion criteria (Table 1 and Figure 7).

### Thorax MRI protocol

All MR examinations of the thorax were performed using a 3.0-T whole-body system (MR750, GE Healthcare, Milwaukee, WI) with a 40-mT/m maximum gradient capability and a standard 8 channel torso coil. Conventional MRI and multi-b value DWI were performed in the same sequence during the same examination. The conventional MRI protocol included respiratory and electrocardiographic gating, T1-weighted spin-echo in the axial plane (repetition time (TR)/echo time (TE), 857 ms/8 ms; matrix size, 288×160; field of view (FOV), 40 cm × 40 cm; number of excitations (NEX), 1; slice thickness, 5 mm; gap, 0.5 mm), T2-weighted fast spin echo in the axial (TR/TE, 4,000 ms/81 ms; matrix size, 288 × 288; FOV, 40 cm × 40 cm; NEX, 1; slice thickness, 5 mm; gap, 0.5 mm) and coronal planes (TR/TE, 4200 ms/78 ms; matrix size, 288×288; field of view, 40 cm × 40 cm; number of excitations, 2; slice thickness, 5 mm; gap, 1.0 mm), and axial T2-weighted turbo spin-echo images with fat suppression (TR/TE, 10,000 ms/182 ms; matrix size, 320×320; field of view, 40 cm × 40 cm; number of excitations, 2; slice thickness, 5 mm; gap, 0.5 mm).

Subsequently, IVIM DWI sequences with 9 b values (0, 25, 50, 100, 150, 200, 500, 800, 1000, and 1200 sec/mm^2^) were performed with a single-shot diffusion-weighted spin-echo echo-planar sequence. We used the respiratory and electrocardiographic triggering and chemical shift selective fat-suppression techniques to reduce artifacts. The lookup table of gradient directions was modified to allow multiple b value measurements in one series. Parallel imaging was used with an acceleration factor of 2. A local shim box covering the whole thorax was applied to minimize susceptibility to artifacts. Other parameters were as follows: TR/TE, 6,000 ms/51 ms; matrix size, 96 × 128; field of view, 40 cm × 40 cm; slice thickness, 5 mm; gap, 0.5 mm. As b values increased, the NEX was also increased from one to six to ensure a good signal-to-noise ratio (SNR). The total scan time was approximately 5 - 6 minutes.

### IVIM DW MRI analysis

According to IVIM, the relationship between signal variation and b values can be expressed using Equation (1) [27]:

$${\text{s}}_{\text{b}}/{\text{s}}_{0}=\text{f}\exp(-\text{b D}*)+(1-\text{f})\exp(-\text{b D})$$ (1)

where S_0_ and S_b_ are the signal intensities at a b value of 0 sec/mm^2^ and each b value other than 0 sec/mm^2^, respectively; D is the true diffusion coefficient that reflects random motion of intra- and intercellular water molecules (slow component of diffusion); f is the fraction of the diffusion linked to microcirculation, and D* is the diffusion parameter representing incoherent microcirculation within the voxel (perfusion-related diffusion, or fast component of diffusion).

Considering that D* is significantly greater than D [19, 27], the influence of D* on signal decay can be neglected for b values greater than 200 sec/mm^2^. Equation (1) can then be simplified, and the estimation of D can be obtained using only b values greater than 200 sec/mm^2^, with a simple linear fit as described in Equation (2):

$${\text{s}}_{\text{b}}/{\text{s}}_{0}=\exp(-\text{b D})$$ (2)

Hence, for high b values (500, 800, 1000, and 1200 sec/mm^2^), S_b_ was first fitted to Equation (2) using a linear model, and the true diffusion coefficient D was calculated. The f and D* values were calculated using a nonlinear regression algorithm based on Equation (1).

The ADC_mb_ value was calculated by fitting the b_0_ image and DW images at each b value other than 0 sec/mm^2^ into the conventional ADC equation (Eq. (3)) [27]:

$${\text{s}}_{\text{b}}/{\text{s}}_{0}=\exp(-\text{b ADC})$$ (3)

### MRI data processing and quantitative analysis

All data were analyzed and processed on a GE ADW4.6 workstation. Routine MRI features were analyzed by a single experienced radiologist, G.-F.L., with 8 years of experience in thoracic MR imaging. The observer was aware that the patients had TETs, but he was blinded to the histological subtypes of the tumors. The routine MR images were analyzed to determine the longest and mean diameter of tumor, shape, homogeneity, and presence of pericardial or pleural effusion. The longest diameter of the tumor was measured at the level where the tumor appeared largest on the cross-sectional image. Based on this, tumors were divided into three groups: < 6.0 cm, 6 - 9 cm, and ≥ 9.0 cm. Mean diameters were calculated as (a + b + c) /3, and the maximal cross-section was used to measure the long and short diameters [11]. Tumor shape was evaluated based on the ratio of the long axis diameter to the short one and was classified as round if the long- to short-axis ratio was less than or equal to 1.5, oval if the ratio was greater than 1.5 but less than 2.0, or plaque if the ratio was greater than or equal to 2.0. The overall signal intensity homogeneity of the tumors was recorded as homogeneous or heterogeneous. The presence of high- and low-signal foci was assessed as a partial signal intensity within a tumor on T2-weighted images [25]. The presence of pericardial or pleural effusions was also evaluated.

The mean values of all IVIM parameters were measured independently by two experienced radiologists, Y.-C.H. and G.-F. L, with 12 and 8 years of experience in thoracic MR imaging, respectively. First, they reviewed the conventional MR images carefully to locate the solid part of each tumor. Next, multi-b-value data were analyzed. A freehand region of interest (ROI) was traced using an electronic cursor and was placed to include the solid tumor elements based on the relative maximum signal intensity on the DW image (bright area, b=1000 sec/mm^2^, as shown in Figure 1B, Figure 2B and Figure 3B) and the relative minimum ADC value in the ADC map (deep-blue area, as shown in Figure 1C, Figure 2C, and Figure 3C), avoiding large vessels and hemorrhagic, calcified, cystic, and necrotic areas. The mean ROI area was 65.2 ± 24.6 mm^2^ (range, 26.0 - 120.0 mm^2^). The IVIM parameter maps were generated automatically (as shown in Figure 1C-E, Figure 2C-E, and Figure 3C-E) and the mean ADC, D, D*, and f values within the ROIs were obtained. The intra-class correlations (ICC) of the two measurements were analyzed to evaluate the consistency of the measurements taken by the two experimenters. The subsequent investigation of diagnostic efficacy was based on the first readings.

### Pathologic diagnosis

Final diagnoses were determined by surgical or puncture biopsy specimens and confirmed by histopathological examination. Pathologic analysis was performed by an expert in the pathology department who was blinded to the clinical and MR findings. Tissue samples obtained from the specimens were processed and stained for hematoxylin and eosin (H&E) using standard protocols. TETs were divided into the following three subgroups based on the criteria of the 2004 World Health Organization (WHO) histological classification guidelines and the Jeong simplified classification of thymic tumors [30, 31]: LRT (types A, AB, and B1), HRT (types B2 and B3), and TC.

TETs were staged according to the following Masaoka-Koga clinical staging system [3]: Stage I: the capsule is intact and microscopic pleural involvement is absent; stage II: the tumor involves surrounding pleura or mediastinal fat, or microscopic pleural involvement is present; stage III: the tumor involves surrounding tissues (pericardium, major blood vessels, or lung); stage IV: the tumor diffuses into the pleura or pericardium (stage IVa), or lymphatic or hematogenous metastasis (stage IVb).

### Statistical analysis

Numerical variables are shown as means with standard deviation. The Kolmogorov–Smirnov (K–S) test was used to assess the normality of data distributions. Binary logistic regression analysis was used to evaluate the effects of multiple parameters in combination. Between-group comparisons of conventional MRI features (including tumor shape, homogeneity, and the presence of pericardial or pleural effusions) were conducted using the chi-square test or Fisher's exact test. Between-group comparisons of tumor mean diameter and maximum diameter were conducted using ANOVAs and Kruskal-Wallis tests, respectively. Differences in the values of the ADC_mb_, D, D*, and f parameters for TETs were compared among LRT, HRT and TC groups using one-way ANOVA, and multiple post hoc comparisons were performed using the Bonferroni (equal variances assumed) and Dunnett's T3 (equal variances not assumed) tests. Differences in the values of the ADC_mb_, D, D*, and f parameters between early (Masaoka stage I and II) and advanced (Masaoka stage III and IV) stage TETs were evaluated using independent sample *t*-tests. Receiver operating characteristic curve (ROC) analyses were performed to determine optimum thresholds for differentiating the defined groups based on various parameters and to calculate sensitivity, specificity, and area under the curve (AUC) values. ICC was used to estimate the agreement between two readings and was interpreted as poor if it was less than 0.4, moderate when it was ≥ 0.4 but < 0.75, and good when it was > 0.75*. P* < 0.05 indicated a statistically significant difference; *P* < 0.05/3 was used for post-hoc tests. IBM SPSS 20.0 software (IBM Corp, Chicago, IL, USA) was used for statistical analysis.

This work was supported by the Tangdu Hospital Development Foundation for Science and Technology Innovation (No. 2015JCYJ010) and Shaanxi Provincial Social Development Science and Technology Project (No. 2016SF-211).

## References

[1] Engels EA (2010). Epidemiology of thymoma and associated malignancies. J Thorac Oncol.

[2] Weis CA, Yao X, Deng Y, Detterbeck FC, Marino M, Nicholson AG, Huang J, Strobel P, Antonicelli A, Marx A (2015). Contributors to the IRD. The impact of thymoma histotype on prognosis in a worldwide database. J Thorac Oncol.

[3] Masaoka A, Monden Y, Nakahara K, Tanioka T (1981). Follow-up study of thymomas with special reference to their clinical stages. Cancer.

[4] Ried M, Marx A, Gotz A, Hamer O, Schalke B, Hofmann HS (2016). State of the art: diagnostic tools and innovative therapies for treatment of advanced thymoma and thymic carcinoma. Eur J Cardiothorac Surg.

[5] Huang J, Detterbeck FC, Wang Z, Loehrer PJ (2010). Standard outcome measures for thymic malignancies. J Thorac Oncol.

[6] Falkson CB, Bezjak A, Darling G, Gregg R, Malthaner R, Maziak DE, Yu E, Smith CA, McNair S, Ung YC, Evans WK (2009). Lung Cancer Disease Site Group of Cancer Care Ontario's Program in Evidence-Based Care. The management of thymoma: a systematic review and practice guideline. J Thorac Oncol.

[7] Okuma Y, Hosomi Y, Watanabe K, Yamada Y, Horio H, Maeda Y, Okamura T, Hishima T (2014). Clinicopathological analysis of thymic malignancies with a consistent retrospective database in a single institution: from Tokyo Metropolitan Cancer Center. BMC Cancer.

[8] Kondo K (2010). Tumor-node metastasis staging system for thymic epithelial tumors. J Thorac Oncol.

[9] Fujii Y (2011). Published guidelines for management of thymoma. Thorac Surg Clin.

[10] Marom EM (2013). Advances in thymoma imaging. J Thorac Imaging.

[11] Hu YC, Wu L, Yan LF, Wang W, Wang SM, Chen BY, Li GF, Zhang B, Cui GB (2014). Predicting subtypes of thymic epithelial tumors using CT: new perspective based on a comprehensive analysis of 216 patients. Sci Rep.

[12] Ozawa Y, Hara M, Shimohira M, Sakurai K, Nakagawa M, Shibamoto Y (2016). Associations between computed tomography features of thymomas and their pathological classification. Acta Radiol.

[13] Sadohara J, Fujimoto K, Muller NL, Kato S, Takamori S, Ohkuma K, Terasaki H, Hayabuchi N (2006). Thymic epithelial tumors: comparison of CT and MR imaging findings of low-risk thymomas, high-risk thymomas, and thymic carcinomas. Eur J Radiol.

[14] Moon JW, Lee KS, Shin MH, Kim S, Woo SY, Lee G, Han J, Shim YM, Choi YS (2015). Thymic epithelial tumors: prognostic determinants among clinical, histopathologic, and computed tomography findings. Ann Thorac Surg.

[15] Razek AA (2012). Diffusion magnetic resonance imaging of chest tumors. Cancer Imaging.

[16] Abdel Razek AA, Khairy M, Nada N (2014). Diffusion-weighted MR imaging in thymic epithelial tumors: correlation with World Health Organization classification and clinical staging. Radiology.

[17] Priola AM, Priola SM, Giraudo MT, Gned D, Fornari A, Ferrero B, Ducco L, Veltri A (2015). Diffusion-weighted magnetic resonance imaging of thymoma: ability of the Apparent Diffusion Coefficient in predicting the World Health Organization (WHO) classification and the Masaoka-Koga staging system and its prognostic significance on disease-free survival. Eur Radiol.

[18] Le Bihan D, Breton E, Lallemand D, Grenier P, Cabanis E, Laval-Jeantet M (1986). MR imaging of intravoxel incoherent motions: application to diffusion and perfusion in neurologic disorders. Radiology.

[19] Le Bihan D, Breton E, Lallemand D, Aubin ML, Vignaud J, Laval-Jeantet M (1988). Separation of diffusion and perfusion in intravoxel incoherent motion MR imaging. Radiology.

[20] Federau C, Maeder P, O’Brien K, Browaeys P, Meuli R, Hagmann P (2012). Quantitative measurement of brain perfusion with intravoxel incoherent motion MR imaging. Radiology.

[21] Hu YC, Yan LF, Wu L, Du P, Chen BY, Wang L, Wang SM, Han Y, Tian Q, Yu Y, Xu TY, Wang W, Cui GB (2014). Intravoxel incoherent motion diffusion-weighted MR imaging of gliomas: efficacy in preoperative grading. Sci Rep.

[22] Woo S, Lee JM, Yoon JH, Joo I, Han JK, Choi BI (2014). Intravoxel incoherent motion diffusion-weighted MR imaging of hepatocellular carcinoma: correlation with enhancement degree and histologic grade. Radiology.

[23] Kang KM, Lee JM, Yoon JH, Kiefer B, Han JK, Choi BI (2014). Intravoxel incoherent motion diffusion-weighted MR imaging for characterization of focal pancreatic lesions. Radiology.

[24] Bourillon C, Rahmouni A, Lin C, Belhadj K, Beaussart P, Vignaud A, Zerbib P, Pigneur F, Cuenod CA, Bessalem H, Cavet M, Boutekadjirt A, Haioun C, Luciani A (2015). Intravoxel incoherent motion diffusion-weighted imaging of multiple myeloma lesions: correlation with whole-body dynamic contrast agent-enhanced MR imaging. Radiology.

[25] Inoue A, Tomiyama N, Fujimoto K, Sadohara J, Nakamichi I, Tomita Y, Aozasa K, Tsubamoto M, Murai S, Natsag J, Sumikawa H, Mihara N, Honda O (2006). MR imaging of thymic epithelial tumors: correlation with World Health Organization classification. Radiat Med.

[26] Tomita M, Matsuzaki Y, Edagawa M, Maeda M, Shimizu T, Hara M, Onitsuka T (2002). Correlation between tumor angiogenesis and invasiveness in thymic epithelial tumors. J Thorac Cardiovasc Surg.

[27] Luciani A, Vignaud A, Cavet M, Nhieu JT, Mallat A, Ruel L, Laurent A, Deux JF, Brugieres P, Rahmouni A (2008). Liver cirrhosis: intravoxel incoherent motion MR imaging--pilot study. Radiology.

[28] Pan CC, Chen WY, Chiang H (2001). Spindle cell and mixed spindle/lymphocytic thymomas: an integrated clinicopathologic and immunohistochemical study of 81 cases. Am J Surg Pathol.

[29] Park HJ, Sung YS, Lee SS, Lee Y, Cheong H, Kim YJ, Lee MG (2017). Intravoxel incoherent motion diffusion-weighted MRI of the abdomen: the effect of fitting algorithms on the accuracy and reliability of the parameters. J Magn Reson Imaging.

[30] Travis WD, Brambilla E, Müller-Hermelink HK, Harris CC (2004). World Health Organization classification of tumours. Pathology and genetics of tumours of the lung, thymus and heart.

[31] Jeong YJ, Lee KS, Kim J, Shim YM, Han J, Kwon OJ (2004). Does CT of thymic epithelial tumors enable us to differentiate histologic subtypes and predict prognosis?. AJR Am J Roentgenol.

